# Robust Road Condition Detection System Using In-Vehicle Standard Sensors

**DOI:** 10.3390/s151229908

**Published:** 2015-12-19

**Authors:** Juan Jesús Castillo Aguilar, Juan Antonio Cabrera Carrillo, Antonio Jesús Guerra Fernández, Enrique Carabias Acosta

**Affiliations:** Department of Mechanical Engineering, Doctor Ortiz Ramos s/n 29071 Malaga, Spain; jcabrera@uma.es (J.A.C.C.); ajguerra@uma.es (A.J.G.F.); eca@uma.es (E.C.A.)

**Keywords:** standard vehicle sensor, friction estimation, optimal slip estimation, normal driving

## Abstract

The appearance of active safety systems, such as Anti-lock Braking System, Traction Control System, Stability Control System, *etc.*, represents a major evolution in road safety. In the automotive sector, the term vehicle active safety systems refers to those whose goal is to help avoid a crash or to reduce the risk of having an accident. These systems safeguard us, being in continuous evolution and incorporating new capabilities continuously. In order for these systems and vehicles to work adequately, they need to know some fundamental information: the road condition on which the vehicle is circulating. This early road detection is intended to allow vehicle control systems to act faster and more suitably, thus obtaining a substantial advantage. In this work, we try to detect the road condition the vehicle is being driven on, using the standard sensors installed in commercial vehicles. Vehicle models were programmed in on-board systems to perform real-time estimations of the forces of contact between the wheel and road and the speed of the vehicle. Subsequently, a fuzzy logic block is used to obtain an index representing the road condition. Finally, an artificial neural network was used to provide the optimal slip for each surface. Simulations and experiments verified the proposed method.

## 1. Introduction

Vehicle active safety systems have experienced continuous progress since the appearance of the first vehicle. The first works in this field were related to Anti-lock Braking Systems (ABS). The development of braking systems to stop the vehicle in the shortest possible distance has been a challenge since automobile manufacturers began to increase the speed and power of their engines. These systems have to maintain the stability and control of the vehicle under any possible situation and road condition. Additionally, instabilities also appear in the vehicle if the opposite process occurs, *i.e.*, wheel slip due to excessive drive torque. These instabilities can affect vehicle passenger safety, especially when the vehicle is driven on a curved trajectory. Stability Control Systems (ESP) and Traction Control Systems (TCS) were developed to avoid the potential risk associated to these situations. These systems, together with the ABS, improve safety in braking, acceleration and cornering processes. Other systems that involve the control of the steering system have also been successfully developed. For example, new driver assistance systems are capable of acting on the steering system and, therefore, control the trajectory of the vehicle thanks to the appearance of modern Electric Power Steering (EPS). Nowadays, most vehicles include active safety systems to avoid accidents or minimize damage during braking, traction and cornering by means of the aforementioned brake, traction and yaw controls.

A new step, which began to develop recently, is autonomous or semi-autonomous driving. This step is a challenge in itself. Several studies are focused on this new field and many companies in the automotive industry are developing prototypes, which must be tested in real environments, such as highways, cities, *etc.* This will be an important step towards the improvement of road safety and to transform the mobility of millions of people.

All previously described active safety systems need some essential information to work properly, which is the road condition on which the vehicle is moving. This information would also be crucial for the “intervention” and “threat assessment” modules in an autonomous or semi-autonomous vehicle [1]. The ‘identification and parameter estimation’ module should provide this information. Obtaining this information when the vehicle is driven in normal conditions, *i.e.*, accelerating, braking or turns without risk of collision, *etc.*, would be very important to prepare the active safety systems of the vehicle to potential danger situations in advance. The information obtained by the vehicle can also be used to inform other drivers about dangerous conditions, so that they can anticipate and change the style of driving in order to improve their own safety.

Several research groups are working on the estimation of the road condition. We can make a division into two main lines of investigation: studies based on the cause and studies based on the effect, commonly called “cause-based” and “effect-based”, respectively. Table 1 shows a summary of different studies found in literature focused on the estimation of road characteristics, divided into the two lines of studies that have previously been mentioned.

**Table 1:** Summary of different methodologies to determine the road condition.

Road Condition Estimation			
**“Cause-based” methods**	**Technique Based on:**	**Type of Sensor:**	**Ref.**
	‘Lubricant’ detection (ice, water, and snow) by means of light absorption	-Optical sensor	[2,3]
	Image analysis	-CCD Camera	[4]
	Backscattering properties	-Radar	[5]
**“Effect-based” methods**	**Technique Based on:**	**Type of Sensor:**	**Ref.**
	Tire deformation	-Strain Gauge -Accelerometer	[6,7]
	Rim deformation	-Strain Gauge	[8]
	Tire noise measurement	-Microphone	[9]
	Tire and wheel speed vibrations.	-ABS wheel speed sensor	[10,11]
	Slip and friction coefficient measurement (“slip-based”)	-ABS wheel speed sensor S -GPS -Fifth Wheel -Accelerometer -Pressure sensor	[12,13,14,15,16,17,18,19,20,21]

Research based on the cause tries to identify factors that affect the friction coefficient and thus predict the road condition using a tire model or an analytic theory. These methods require the use of special sensors such as optical sensors, radars or cameras. Among the main disadvantages of these methods are: they require the use of sensors that are not part of the standard equipment of vehicles; the need to perform extensive training to obtain good results; and, finally, they often fail under exceptional conditions that have not been trained or that the sensor cannot detect. Some of the main advantages are that they do not need any excitation to determine the road condition; that is to say, they can provide the road condition even when the vehicle is moving in steady conditions, without accelerating or decelerating.

**Figure 1:**
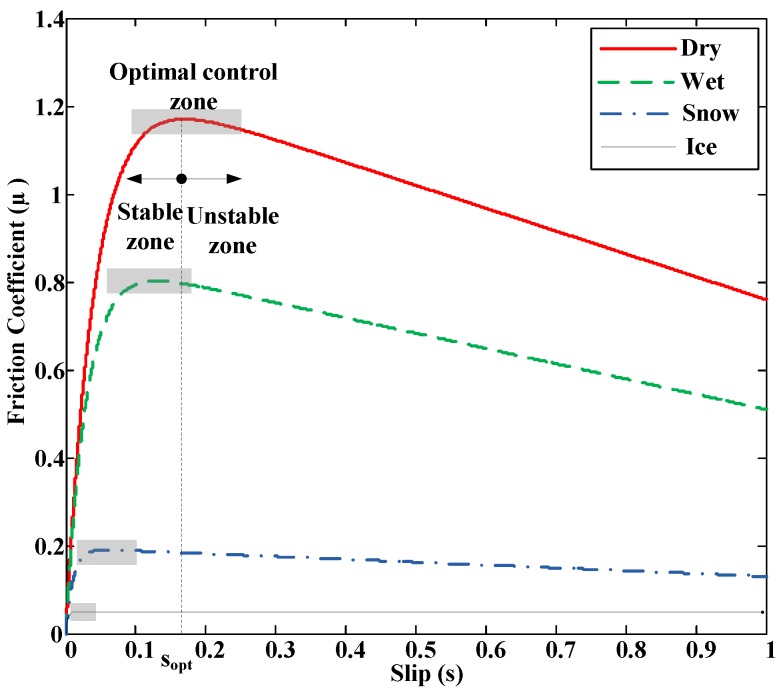
Adhesion curves for different surfaces with optimal control areas.

Research based on the effect focuses on the effects that appear due to tire-road friction. Therefore, they require an excitation (acceleration or braking) to make those effects perceptible. These investigations include methods that employ tire and rim deformation, noise, vibration or slip to detect the road condition. Methods based on the measurement of tire deformation use sensors embedded in the tires to measure deformation as a function of the angular position of the tire. The condition of the road on which the vehicle circulates is inferred from this information. Noise methods perform tire noise measurements using a microphone to detect tire behavior and, from this data, determine the friction in the tire-road contact. The main disadvantage of these two methods is that they require the use of sophisticated sensors. Vibration methods determine the resonance frequency of the angular velocity of the wheel from its frequency spectrum. They are able to obtain information about the road condition from the study of the value of the resonance frequency. The main advantage of these methods is that they use the wheel angular velocity sensor, which is a standard ABS component installed in most vehicles. Among the disadvantages, we can mention the influence of several factors in the determination of the resonance frequency and poor accuracy of the angular velocity measurement. Finally, the slip-based methods, which are the most used, are based on obtaining the wheel slip and the friction coefficient to determine the road condition. They investigate the differences between the adhesion curves of the tire-road contact on different surfaces (see Figure 1). In these curves it can be observed that for large slips (unstable region) it is relatively easy to determine the road condition if we know the coefficient of friction (µ) and slip. However, the problem is in the area where s < 0.1, low excitation, such as slips caused by low accelerations or decelerations. These low levels of slips are typically produced during normal driving. In this area the behavior is linear and there is little difference between different surfaces.

Therefore, the development of systems to obtain real time estimates of the road condition, both in four-wheeled vehicles and motorcycles, is of great interest. This paper presents a method to detect the road condition and the optimal slip (s_opt_) using ABS and ESP standard sensors installed in vehicles. Optimal slip is defined as the slip where the maximum adherence occurs for a specific surface (see, Figure 1). The algorithm to estimate the road condition and the optimal slip is tested under normal driving situations, *i.e.*, low slip, and in extreme driving situations, high slip.

The contributions of this work with respect to existing approaches are:

(I) The development of an estimation algorithm based on Extended Kalman Filter to estimate tire forces and vehicle speed. These estimates are used to obtain the tire-road friction coefficient and the slip.
(II) To estimate the road condition using the obtained friction coefficient, the slip and the rate of change of friction coefficient *vs.* slip. The estimation process is carried out using a fuzzy logic based block. The use of fuzzy logic is suitable for its ability to adapt to nonlinear processes and when there is much uncertainty, obtaining results that are in accordance with reality.
(III) The comparison of two algorithms for estimating the road condition. The first algorithm uses the estimated friction coefficient and slip to provide an index indicative of the adherence of the road. The second algorithm uses the friction coefficient, the slip and the rate of change of friction coefficient *vs.* the slip. It is shown that the second algorithm detects changes in road conditions faster and provides values that are more accurate.
(IV) Finally, to determine the optimal slip by means of artificial neural networks. The network training is performed using the adhesion curves provided by a tire-road contact model. Optimum slip is a key parameter that can be used to improve vehicle performance and safety in traction and braking processes and cornering.

Section 2 describes vehicle and wheel models, used in estimation processes. Section 3 presents a method to estimate the actual vehicle speed and the forces of contact between the wheel and road based on a Kalman filter. A Fuzzy-Logic-based control block for the detection of the road condition and an Artificial Neural Network (ANN) to obtain the optimal slip are described in Section 4 and Section 5 respectively. Simulations results are included in Section 6. Section 7 outlines a sensorized vehicle used to test the new system experimentally and presents the results obtained in real tests. Finally, Section 8 concludes this paper.

## 2. Modeling

### 2.1. Vehicle Model

**Figure 2:**
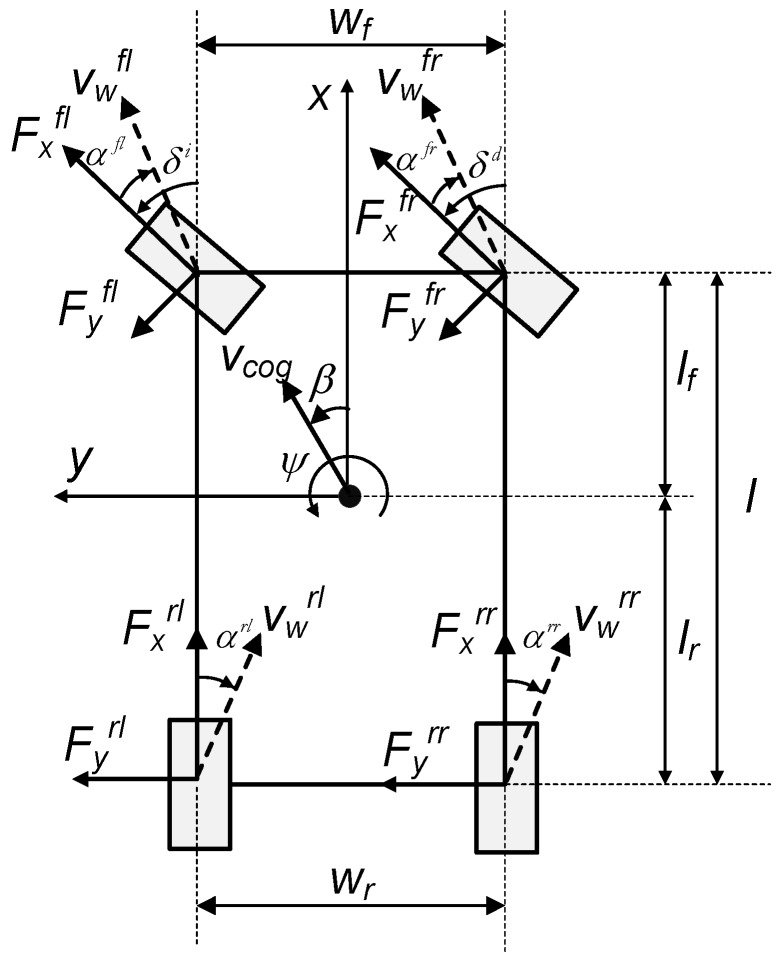
Four-wheel longitudinal-lateral model.

This section describes the mathematical model of the vehicle used throughout the paper (Figure 2) [22]. The following considerations are taken into account when developing the model:

- The vehicle has a longitudinal symmetry plane.
- The origin of the mobile coordinate system coincides with the center of gravity of the vehicle.
- Pitch and roll motion are not taken into account.
- Traction and steering systems only act on the front wheels.

Parameters and variables used in this model are shown in Table 2.

**Table 2:** Parameters in the four-wheel longitudinal-lateral model.

	Description		Description
M	Vehicle mass	Fx^fr^	Longitudinal force in the front axle, right wheel
I_z_	Moment of inertia in the z-axis	Fx^fl^	Longitudinal force in the front axle, left wheel
l_f_	Distance between the center of gravity and the front axle	Fx^rr^	Longitudinal. force in the rear axle, right wheel
l_r_	Distance between the center of gravity and the rear axle	Fx^rl^	Longitudinal force in the rear axle, left wheel
L	Wheelbase	Fy^fr^	Lateral force in the front axle, right wheel
h	Height of the cog	Fy^fl^	Lateral force in the front axle, left wheel
w_f_	Front axle track	Fy^rr^	Lateral force in the rear axle, right wheel
w_r_	Rear axle track	Fy^rl^	Lateral force in the rear axle, left wheel
α^fr^	Slip angle of the front right wheel	Fz^fr^	Vertical load in the front axle, right wheel
α^fl^	Slip angle of the front left wheel	Fz^fl^	Vertical load in the front axle, left wheel
α^rr^	Slip angle of the rear right wheel	Fz^rr^	Vertical load in the rear axle, right wheel
α^rl^	Slip angle of the rear left wheel	Fz^rl^	Vertical load in the rear axle, left wheel
β	Vehicle slip angle	µ_x_^fr^	Longitudinal adhesion coefficient, front right wheel.
δ^r^	Steering angle of the right wheel	µ_x_^fl^	Longitudinal adhesion coefficient, front left wheel.
δ^l^	Steering angle of the left wheel	µ_x_^rr^	Longitudinal Adhesion coefficient. rear right wheel
v_cog_	Absolute velocity of the center of gravity	µ_x_^rl^	Longitudinal adhesion coefficient, rear left wheel
ψ	Turn about the z-axis (yaw)	µ_y_^fr^	Lateral adhesion coefficient, front right wheel
ρ	Air density	µ_y_^fl^	Lateral adhesion coefficient, front left wheel
C_x_	Drag Coefficient	µ_y_^rr^	Lateral adhesion coefficient, rear right wheel
F_x_^a^	Drag Force	µ_y_^rl^	Lateral adhesion coefficient, rear left wheel

The equations that define longitudinal, lateral and yaw motions are:

(1) $$M\cdot \left(\ddot{x}-\dot{y}\cdot \dot{\psi }\right)=F_{x}^{fl}\cdot \cos\delta^{l}-F_{y}^{fl}\cdot \sin\delta^{l}+F_{x}^{fr}\cdot \cos\delta^{r}-F_{y}^{fr}\cdot \sin\delta^{r}+F_{x}^{rl}+F_{x}^{rr}-F_{x}^{a}$$

(2) $$M\cdot \left(\ddot{y}+\dot{x}\cdot \dot{\psi }\right)=F_{y}^{fl}\cdot \cos\delta^{l}+F_{x}^{fl}\cdot \sin\delta^{l}+F_{y}^{fr}\cdot \cos\delta^{r}+F_{x}^{fr}\cdot \sin\delta^{r}+F_{y}^{rl}+F_{y}^{rr}$$

(3) $$\begin{array}{cc} I_{z}\cdot \ddot{\psi }= & \left(F_{y}^{fl}\cdot \cos\delta^{l}+F_{x}^{fl}\cdot \sin\delta^{l}\right)\cdot l_{f}+\left(F_{y}^{fr}\cdot \cos\delta^{r}+F_{x}^{fr}\cdot \sin\delta^{r}\right)\cdot l_{f}-F_{y}^{fl}\cdot l_{r}-F_{y}^{rl}\cdot l_{r}-\\ & -\frac{1}{2}\cdot \left(F_{x}^{fl}\cdot \cos\delta^{l}-F_{y}^{fl}\cdot \sin\delta^{l}\right)\cdot w_{f}+\frac{1}{2}\cdot \left(F_{x}^{fr}\cdot \cos\delta^{r}-F_{y}^{fr}\cdot \sin\delta^{r}\right)\cdot w_{f}-\\ & -\frac{1}{2}\cdot F_{x}^{rl}\cdot w_{r}+\frac{1}{2}\cdot F_{x}^{rr}\cdot w_{r} \end{array}$$

Longitudinal and lateral forces are given by:

(4) $$\begin{array}{ccc} F_{x}^{ij}=\mu_{x}^{ij}\cdot F_{z}^{ij} & & F_{y}^{ij}=\mu_{y}^{ij}\cdot F_{z}^{ij} \end{array}$$

In this work, vertical forces are computed using longitudinal and lateral accelerations as it is described in [23]. A linear model was used due to its good compromise between accuracy and simplicity. While a more complete vehicle model may be more accurate, its programming within a control loop reduces the frequency at which this can be run.

(5) $$\begin{array}{l} F_{z}^{fr}=M\cdot g\cdot \frac{l_{r}}{2\cdot l}-\frac{M\cdot h}{2\cdot l}\cdot a_{x}+\frac{M\cdot h\cdot l_{r}}{w_{d}\cdot l}\cdot a_{y}\\ F_{z}^{fl}=M\cdot g\cdot \frac{l_{r}}{2\cdot l}-\frac{M\cdot h}{2\cdot l}\cdot a_{x}-\frac{M\cdot h\cdot l_{r}}{w_{d}\cdot l}\cdot a_{y}\\ F_{z}^{rr}=M\cdot g\cdot \frac{l_{f}}{2\cdot l}+\frac{M\cdot h}{2\cdot l}\cdot a_{x}+\frac{M\cdot h\cdot l_{r}}{w_{t}\cdot l}\cdot a_{y}\\ F_{z}^{fl}=M\cdot g\cdot \frac{l_{f}}{2\cdot l}+\frac{M\cdot h}{2\cdot l}\cdot a_{x}-\frac{M\cdot h\cdot l_{r}}{w_{t}\cdot l}\cdot a_{y} \end{array}$$

where *a_x_* and *a_y_* are the longitudinal and lateral accelerations, respectively:

(6) $$a_{x}=\ddot{x}-\dot{y}\cdot \dot{\psi }$$

(7) $$a_{y}=\ddot{y}+\dot{x}\cdot \dot{\psi }$$

Slip angles, (*α_fr_*, *α_fl_*, *α_rr_*, *α_rl_*), of the four wheels are calculated as follows:

(8) $$\begin{array}{ccc} \alpha^{fr}=\delta^{f}-\tan^{-1}\left(\frac{\dot{y}+l_{f}\cdot \dot{\psi }}{\dot{x}+\frac{w_{f}}{2}\cdot \dot{\psi }}\right) & & \alpha^{fl}=\delta^{l}-\tan^{-1}\left(\frac{\dot{y}+l_{f}\cdot \dot{\psi }}{\dot{x}-\frac{w_{f}}{2}\cdot \dot{\psi }}\right)\\ \alpha^{rr}=-\tan^{-1}\left(\frac{\dot{y}-l_{r}\cdot \dot{\psi }}{\dot{x}+\frac{w_{r}}{2}\cdot \dot{\psi }}\right) & & \alpha^{rl}=-\tan^{-1}\left(\frac{\dot{y}-l_{r}\cdot \dot{\psi }}{\dot{x}-\frac{w_{r}}{2}\cdot \dot{\psi }}\right) \end{array}$$

The slip angle of the vehicle (*β*) is computed from the following equation:

(9) $$\beta =\tan^{-1}\left(\frac{\dot{y}}{\dot{x}}\right)$$

### 2.2. Wheel Model 

The equation which models wheel behavior is:

(10) $$I_{r}\cdot {\dot{w}}_{r}=T_{T}-T_{B}-M_{R}-F_{x}\cdot R$$

where *I_r_* is the moment of inertia of the wheel, *ω_r_* is the wheel angular velocity, *F_x_* is the longitudinal force, *T_T_* and *T_B_* are de traction and brake torques respectively, *M_R_* is the rolling resistance torque, *R* is the wheel radius, *µ* is the adhesion coefficient and *F_z_* is the vertical load. With:

(11) $$F_{x}=\mu \cdot F_{z}$$

The Burckhardt model [24] was used to obtain tire forces in simulations. This model yields the longitudinal and lateral friction coefficients depending on the slip and slip angle for different surface types.

## 3. Adhesion Coefficients, Velocity and Slip Angle Estimation

**Figure 3:**
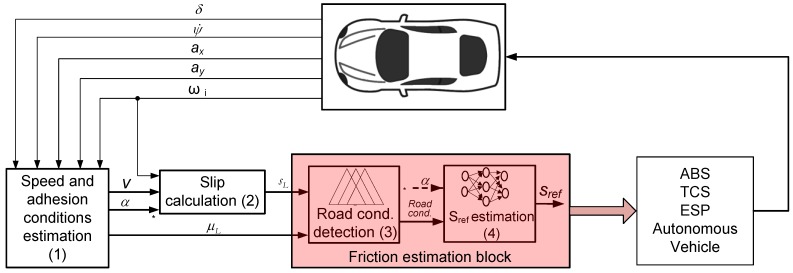
Block diagram of the friction estimation algorithm.

The control loop to obtain the friction characteristics is shown in Figure 3. The four blocks are applicable to any ABS, TCS, ESP system configuration and autonomous vehicle, since these systems need road adhesion characteristics to work properly and more efficiently.

The first block of the control algorithm estimates the value of the tire-ground adhesion coefficient of each wheel, the velocity of the vehicle and the slip angle using the vehicle model described in the previous section and the measured variables from vehicle sensors. 

Kalman filter allows estimating the state of a linear dynamic system perturbed by white noise using measurements that are linear functions of the system state but corrupted by white noise. In this work, an EKF is used for parameter estimation purposes making use of the equations of the longitudinal-lateral vehicle model [25,26,27,28]. 

The measured variables are *ω*, *a_y_*, *a_x_*,$\dot{\;\psi }$, and *δ*, where *ω* represents the angular velocity of the wheels, *a_x_* and *a_y_* are the longitudinal and lateral acceleration, respectively, $\dot{\psi }$ is the yaw rate and *δ* is the steering angle, which is considered to be equal for both front wheels. The estimated variables are *v_x_*, *v_y_*, $\dot{\psi }$, *a_x_*, *a_y_*, *F_x_*, and *F_y_*, where *v_x_* and *v_y_* are the longitudinal and lateral velocities and *F_x_* and *F_y_* are the longitudinal and lateral forces in the tire-ground contact patch, respectively. Some measured variables also appear as estimated variables. In such cases, the estimator is used as a noise reduction filter. Finally, longitudinal and lateral adhesion coefficients, expressed as $\mu_{L}$ and $\mu_{S}$, are obtained from the estimated horizontal and lateral forces and the calculated vertical loads.

**Figure 4:**
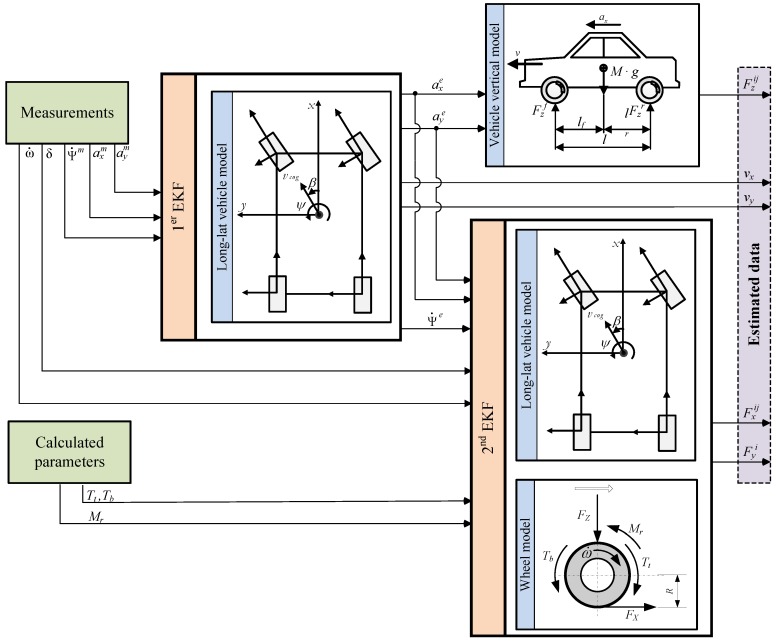
Estimation algorithm scheme.

Figure 4 summarizes the functioning of the algorithm, which is explained next. The algorithm starts by computing a series of variables such as the braking torque (*T_b_*), the traction torque (*T_t_*) that can be obtained from engine and brake system models, and the rolling resistance (*M_r_*). These variables and the measured variables are the inputs to the EKF estimation algorithm. At the same time, longitudinal and lateral accelerations are the inputs to the vertical vehicle model, which yields the vertical load on each wheel. The algorithm makes use of the wheel and vehicle models to provide an estimate of the longitudinal and lateral loads. These are necessary to compute the adhesion coefficients, slip angle and velocity.

Two estimation algorithms are implemented in series. The first one has the acceleration and yaw rate as inputs. The outputs are the longitudinal and lateral velocities and the filtered values of the accelerations and yaw rate. The second algorithm has the filtered accelerations, the steering angle and the angular velocity of the wheels as inputs and yields the horizontal forces. This approach requires a lower computational cost and smaller Kalman filter matrices than a single algorithm comprehending all the parameters. Moreover, better force estimates are obtained since filtered acceleration and yaw rate are used in the second estimation algorithm. 

The state vector in the first estimation algorithm is

(12) $$x(t)={\left[v_{x},v_{y},\dot{\psi },a_{x},a_{y}\right]}^{T}$$

and the measurement vector is

(13) $$z(t)={\left[a_{x}^{m},a_{y}^{m},{\dot{\psi }}^{m}\right]}^{T}$$

where superscript “m” indicates a measured value by the corresponding sensor. 

The state and measurement vector in the second estimation algorithm are:

(14) $$x(t)={\left[F_{x}^{fr},F_{x}^{fl},F_{y}^{f},F_{x}^{rr},F_{x}^{rl},F_{y}^{r}\right]}^{T}$$

(15) $$z(t)={\left[a_{x}^{e},a_{y}^{e},{\ddot{\psi }}^{e},{\dot{\omega }}^{fr,m},{\dot{\omega }}^{fl,m},{\dot{\omega }}^{rr,m},{\dot{\omega }}^{rl,m},\delta \right]}^{T}$$

where *F* represents the value of the force to be estimated. Random walk models were used to obtain the forces in the second Kalman estimator ([17] and [29]). Random walks are defined when there are no dynamic equations for the parameters to be estimated. First order random walks assume the first derivative of the value to be zero. Therefore, the horizontal forces random walks are $F_{x}^{ij}\left(t\right)=F_{x}^{ij}\left(t-1\right)$ and $F_{y}^{i}\left(t\right)=F_{y}^{i}\left(t-1\right)$, where subscript *i* is used for the front and rear axle and subscript *j* for the left and right wheels. Only one lateral force per vehicle axle is estimated. This is due to the fact that there are only three model equations that relate lateral forces and measurements. Therefore, the estimator cannot estimate four lateral forces adequately.

Slip coefficients are computed from the equations included in Table 3. As it can be noticed, these coefficients depend on the tire rolling speed (*v_R_*), the velocity of the center of the contact surface of the tire (*v_w_*) and the slip angle (*α*). The rolling speed of the tire depends on the tire radius (*R*), a parameter that is generally taken as constant or dependent on the vertical load and the wheel angular velocity ($\omega_{r}$), which is measured from the angular sensors on the wheel (see Equation (8)). 

**Table 3:** Slip definition.

	Braking $\left(v_{R}\cos\alpha \leq v_{w}\right)$	Traction $\left(v_{R}\cos\alpha >v_{w}\right)$
**Longitudinal slip, S_L_**	$s_{L}=\frac{v_{R}\cdot \cos\alpha -v_{w}}{v_{w}}$	$s_{L}=\frac{v_{R}\cdot \cos\alpha -v_{w}}{v_{R}\cdot \cos\alpha }$
**Lateral slip, S_S_**	$s_{S}=\frac{v_{R}\cdot \sin\alpha }{v_{w}}$	$s_{S}=\tan\alpha$

The velocity of the center of the tire contact surface ($v_{w,ij}$) depends on the absolute velocity of the vehicle ($v_{cog}$), the yaw rate ($\dot{\psi }$) and the distance from the wheel to the center of gravity ($\Delta r_{ij}$) (see Equation (17)). Last, the slip angles are a function of the lateral and longitudinal velocities of the vehicle, the steering angle (*δ*) and the yaw rate (Equation (8)).

(16) $$v_{R}=\omega_{r}\cdot R$$

(17) $$v_{w,ij}=v_{cog}\mp \dot{\psi }\Delta r_{ij}$$

In this paper, the tire radius is calculated from:

(18) $$R=R_{unloaded}-\frac{Fz}{K_{t}}$$

where *R_unloaded_* is the unloaded tire radius, *F_z_* is the estimated vertical force on that wheel and *K_t_* is the tire vertical stiffness.

## 4. Road Condition Detection

A fuzzy logic block is used to detect the road condition once the adhesion coefficient, vehicle velocity and wheel slip have been estimated. This block takes the longitudinal slip, adhesion coefficient and the rate of change of adhesion coefficient *vs.* slip as inputs. It produces a value as output that indicates the road condition the vehicle is running on. This output is called Road Condition Index (RCI). Therefore, this index can be used to monitor the road condition. The higher the index is, the higher the adherence of the road. RCI is within range 0–1.2, where 1.2 represents the road condition with the highest adhesion coefficient and 0 represents the lowest one. Road condition detection without using additional sensors is challenging due to inherent nonlinearities in the problem. Sensor noise and the need for fast detection also make it difficult to obtain reliable estimates to be used in vehicle control systems. Therefore, fuzzy logic is an appropriate tool to deal with such systems thanks to its aptitude to deal with nonlinearities and uncertainties.

Five membership functions are defined for the input linguistic variable friction and three for the longitudinal slip and dμ/ds linguistic variables. Eight membership functions are associated to the output linguistic variable RCI. The membership functions are defined below (see Table 4):

**Table 4:** Membership functions.

Friction Coefficient		Slip		dμ/ds		RCI	
VL	Very Low	VL	Very Low	Ldμ/ds	Low	ZFR	Zero friction road
L	Low	M	Medium	Mdμ/ds	Medium	VSFR	Very small friction road
M	Medium	H	High	Hdμ/ds	High	SFR	Small friction road
H	High					MFR	Medium friction road
VH	Very High					LFR	Large friction road
						VLFR	Very Large friction road
						HFR	High friction road
						EFR	Extreme friction road

**Figure 5:**
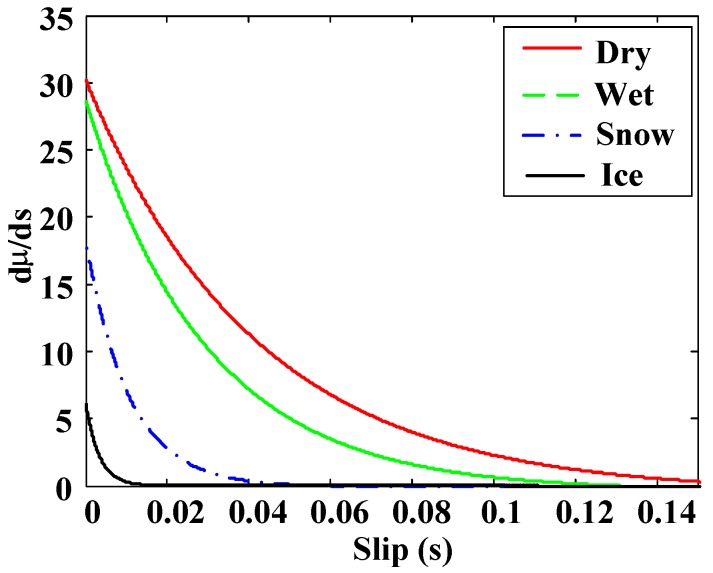
dµ/ds *vs.* s curves for different surfaces.

The following analysis was followed to define the rules: if the slip is small, the adhesion of the road cannot be determined without the slope of the µ–s curve. This input improves the performance of the block in very low slip conditions. It can be seen, from the observation of the adhesion curves, that the higher the adherence of the road is, the higher the slope of the μ–s curve is (Figure 5). This means that for low adhesion surfaces, the slope of the µ–s trajectory is small when the slip is small. On the contrary, if the slope of the µ–s trajectory is high when the slip is small, it indicates that the vehicle is moving on a high adhesion surface.

For medium and high slips, the road condition can be determined using only the friction coefficient and the slip. If the friction coefficient is high, it indicates that the surface is highly adherent. For medium slip values, the output of the block increases with the estimated friction coefficient. Finally, for high slip values, the output also increases with the adherence coefficient reaching slightly higher values than the ones defined for medium values. A second fuzzy block to estimate the road condition is designed. This second block does not take dµ/ds into account to determine the road condition. Therefore, the following rules are defined for the fuzzy inference systems (Table 5).

**Table 5:** Fuzzy rules.

Rule Number	Fuzzy Block with dμ/ds				Fuzzy Block without dμ/ds		
	Friction Coefficient	Slip	dμ/ds	RCI	Friction Coefficient	Slip	RCI
1	VL	VL	Ldμ/ds	ZFR	VL	VL	SFR
2	VL	VL	Mdμ/ds	VSFR	VL	M	VSFR
3	VL	VL	Hdμ/ds	VLFR	VL	H	ZFR
4	VL	M	---	ZFR	L	VL	LFR
5	VL	H	---	VSFR	L	M	SFR
6	L	VL	Ldμ/ds	SFR	L	H	MFR
7	L	VL	Mdμ/ds	LFR	M	VL	VLFR
8	L	VL	Hdμ/ds	VLFR	M	M	MFR
9	L	M	---	SFR	M	H	MFR
10	L	H	---	MFR	H	VL	HFR
11	M	VL	Ldμ/ds	LFR	H	M	VLFR
12	M	VL	Mdμ/ds	HFR	H	H	HFR
13	M	VL	Hdμ/ds	HFR	VH	VL	EFR
14	M	M	---	MFR	VH	M	HFR
15	M	H	---	MFR	VH	H	EFR
16	H	VL	Ldμ/ds	VLFR			
17	H	VL	Mdμ/ds	HFR			
18	H	VL	Hdμ/ds	EFR			
19	H	M	---	VLFR			
20	H	H	---	HFR			
21	VH	VL	Ldμ/ds	EFR			
22	VH	VL	Mdμ/ds	EFR			
23	VH	VL	Hdμ/ds	EFR			
24	VH	M	---	HFR			
25	VH	H	---	EFR			

Each row of Table 5 has the following meaning: IF Friction coefficient IS (value in column 2 or 6) AND slip IS (value in column 3 or 7) AND dμ/ds IS (value in column 4) THEN RCI IS (value in column 5 or 8). Twenty-five rules are defined for the first block and fifteen for the second one. dμ/ds is not taken into account when the slip is medium or high because no significant contribution is provided to the estimations since the road condition can accurately be estimated using only the friction coefficient and the slip.

**Figure 6:**
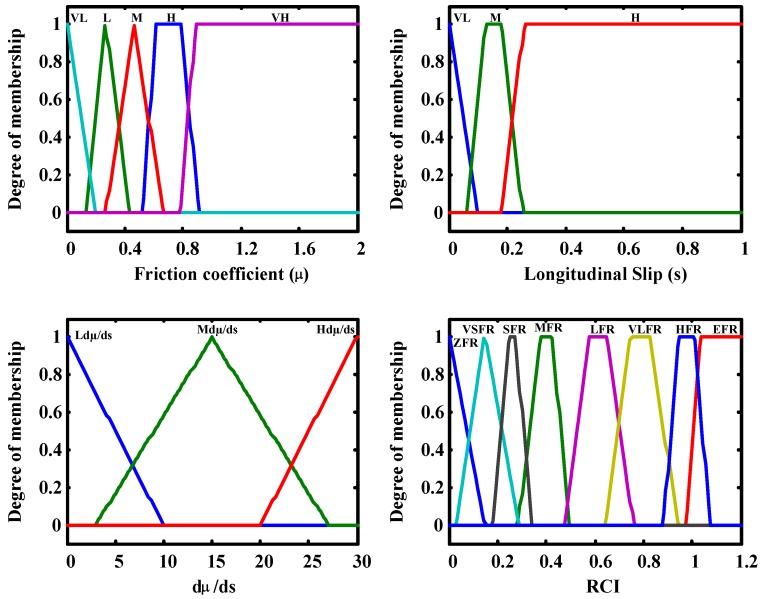
Membership functions of the Fuzzy Road condition estimation block.

**Figure 7:**
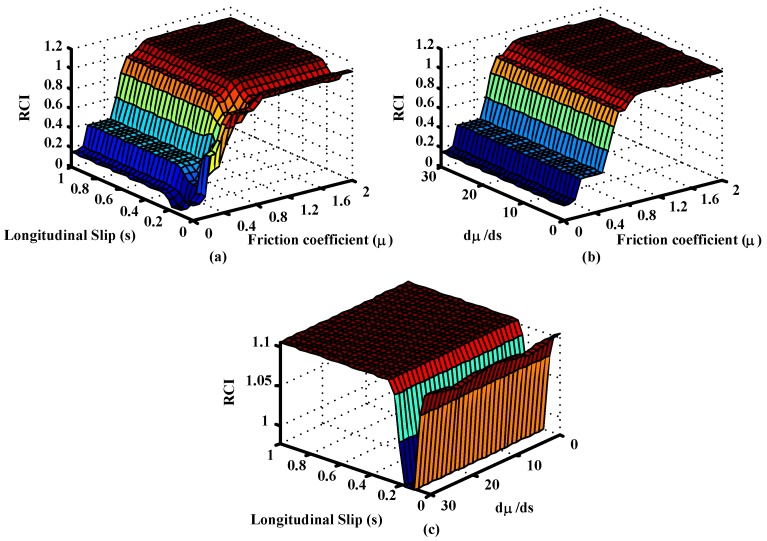
Output surfaces of the Fuzzy Road condition estimation block with dμ/ds. (**a**) RCI *vs.* Longitudinal Slip and Friction coefficient (**b**) RCI *vs.* dμ/ds and Friction coefficient (**c**) RCI *vs.* Longitudinal Slip and dμ/ds.

Membership functions and the surface generated by the fuzzy inference system are shown in Figure 6 and Figure 7, respectively.

**Figure 8:**
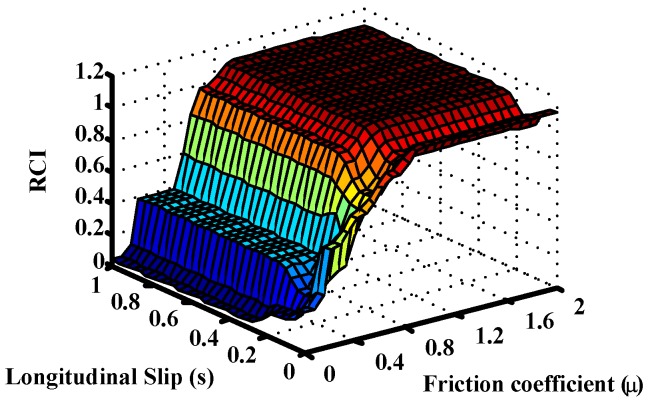
Output surfaces of the Fuzzy Road condition estimation block without dμ/ds.

The following figure shows the surface generated by the fuzzy road condition estimation block (Figure 8). The differences between Figure 7a and Figure 8 in the region of low slip and low friction coefficient are due to the definitions of the rules for the controls with and without dμ/ds. These rules are set out in Table 5. As shown, control without dμ/ds uses the coefficient of friction to provide the Road Condition Index when the slip is small. The control with dμ/ds uses this information along with the coefficient of friction to estimate the Road Condition Index when the slips are small (VL). Therefore, the surfaces generated in both figures are different in the low slips region. 

## 5. Optimal Slip Estimation

Both the longitudinal and lateral adhesion of the tire for a given surface depend mainly on the slip. Tire/road friction curves show a maximum adhesion value for a given slip, which is called optimal slip (s_opt_) and then a progressive decrease until total slip is reached. Therefore, for a given surface, the optimal slip is defined as the slip rate that produces the maximum longitudinal friction coefficient.

In general, control algorithms try to keep the slip as close as possible to the optimal slip (shaded area in Figure 1). The optimal slip differentiates two working areas: a stable one and an unstable one. In the stable region, an increase in the traction or braking torque produces an increment in the slip and, with it, the adhesion coefficient increases, which means that the longitudinal force also increases, causing a higher acceleration or deceleration. In the unstable working zone, increasing the traction or braking torque causes greater sliding and thus, lower adhesion, which means lower acceleration rates or larger braking distances. Beyond the optimal slip, the wheels tend to lock quickly unless the applied traction or braking torque is reduced.

In this paper, the optimal slip is obtained by means of an artificial neural network (ANN). The inputs are the road condition and the slip angle. The first step was to normalize the different road conditions provided by the Burckhardt model within 0 and 1. Each road condition was assigned a value proportional to its maximum longitudinal friction coefficient. Thus, a road with high adhesion received a value of 1 and a road with very low adhesion was given a value close to 0. The rest of surfaces were given a normalized value proportional to their maximum longitudinal friction coefficient (Table 6), where V_m_ is the maximum value of the curve and V_n_ is the corresponding normalized value.

**Table 6:** Maximum and normalized values.

Surface	Vm	Vn
**Dry asphalt**	1.170	1.000
**Wet asphalt**	0.801	0.685
**Dry concrete**	1.090	0.932
**Wet cobblestone**	0.380	0.325
**Snow**	0.190	0.163
**Ice**	0.020	0.017

A three-layer ANN was implemented in MATLAB^®^ having nine neurons in the first layer, a second hidden layer with three neurons and one neuron in the output layer. The transfer functions used were sigmoid in the first two layers and linear in the third one. The Levenberg-Marquardt back propagation method was used to train the network.

**Figure 9:**
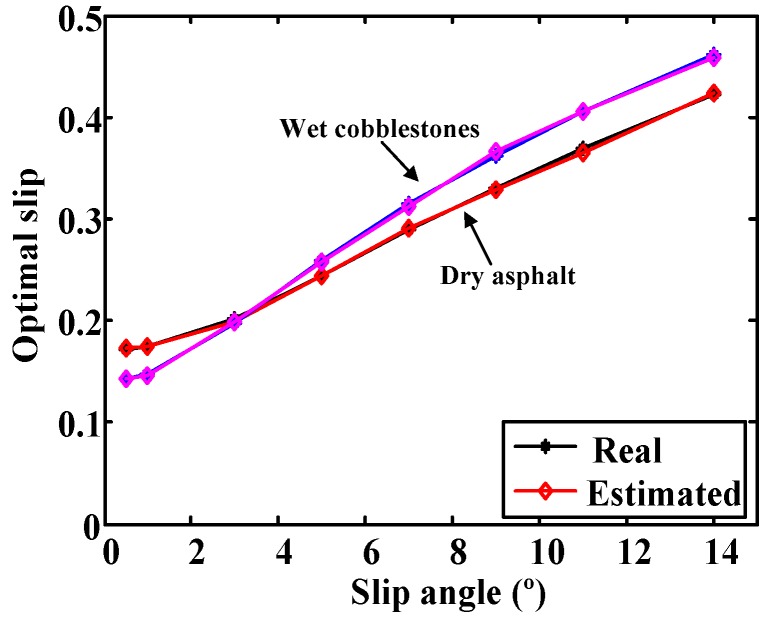
Comparison between the real optimal slip and the optimal slip provided by the ANN.

The curves provided by the Burckhardt model with slip angles of 0, 2, 4, 6, 8, 12 and 16° were used to train the network. The input data were the type of surface and the slip angle and the output was the slip value that provides maximum longitudinal adhesion for each surface and angle. Figure 9 shows the output of the ANN for dry asphalt and wet cobblestone with angles 0.5, 1, 3, 5, 7, 9, 11, and 14°. Optimal slips from the Burckhardt model are included for comparison purposes. It can be seen that the output of the ANN fits perfectly in the real optimal slip for slip angles not included in the training process.

## 6. Simulations

The road condition estimation algorithm was programmed in Simulink^®^. CarSim^®^ was incorporated as an S-function to simulate vehicle behavior. Carsim^®^ provided the measures obtained in the vehicle in each test. 

**Figure 10:**
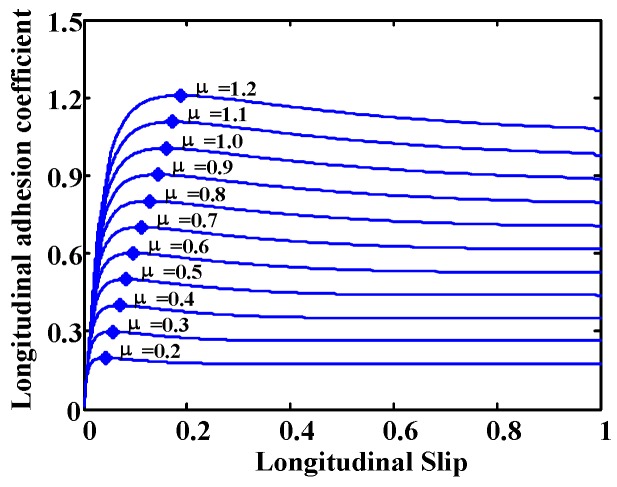
Approximated CarSim^®^ adherence *vs.* slip curves.

Different types of surfaces were used in the simulations. Surfaces are defined in CarSim^®^ by a parameter indicative of its adherence. For the sake of clarity, Figure 10 shows the adherence *versus* slip curves for several values of this parameter. However, these curves are approximate since actual adhesion depends on, among other variables, velocity, slip angle and type of tire.

Zero–mean white noises were added to the simulated measurements, including accelerations and wheel speed. Sensor noise covariances were obtained from experimental tests with the sensors installed on the vehicle. Process noise covariances were determined and optimized using Genetic Algorithms. 

### 6.1. Straight Line Simulation

In this simulation, the vehicle starts on a high-adhesion surface (μ = 1) and, after travelling 20 m on it, it moves to a low-adherence surface (μ = 0.4) for 40 m. Finally, the vehicle moves back to the high-adherence surface. This simulation is included to test the ability of the estimation algorithm to adapt its outputs to sudden changes in adhesion conditions.

**Figure 11:**
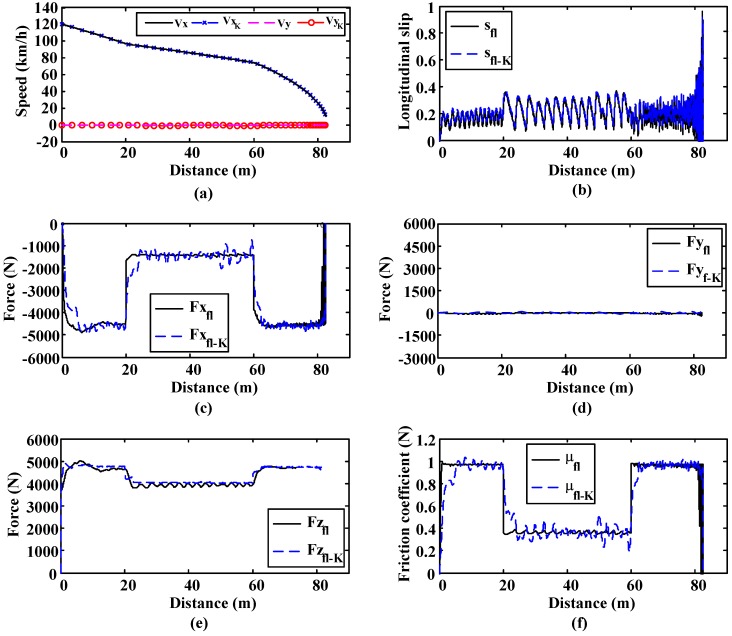
Straight line simulation. Surface transition: 20 m. on μ = 1, 40 m. on μ = 0.4 and 40 m. on μ = 1. (**a**) Longitudinal and lateral speed; (**b**) Longitudinal slip; (**c**) Longitudinal forces; (**d**) Lateral forces; (**e**) Vertical forces; (**f**) Friction coefficient.

As can be seen in Figure 11, the estimation algorithm yields accurate estimates of speed and friction coefficient even when low slips occur. Horizontal (Fx_fl-K_), vertical (Fz_fl-K_) and lateral force (Fy_f-K_) estimates are also included and compared to the real values. It can be verified that in all cases the estimates matches the real values perfectly.

### 6.2. Curve Simulation

Cornering tests were performed to verify the performance of the road estimation algorithm under such conditions. This simulation is included to show how the estimation algorithm can estimate lateral forces and slip angles correctly when the vehicle is moving round a bend with a 500 m-radius. 

**Figure 12:**
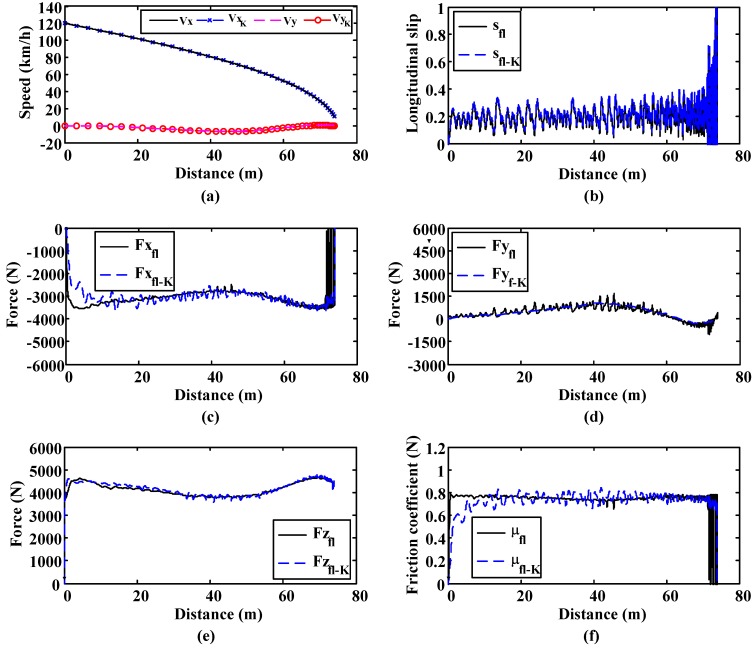
Curve simulation. Surface μ = 0.8. 500 m. radius curve. (**a**) Longitudinal and lateral speed; (**b**) Longitudinal slip; (**c**) Longitudinal forces; (**d**) Lateral forces; (**e**) Vertical forces; (**f**) Friction coefficient.

The road condition is kept constant in this simulation, being equal to 0.8. Similarly to the previous simulation, it is shown that all the parameters are accurately estimated during the whole simulation (Figure 12). 

**Figure 13:**
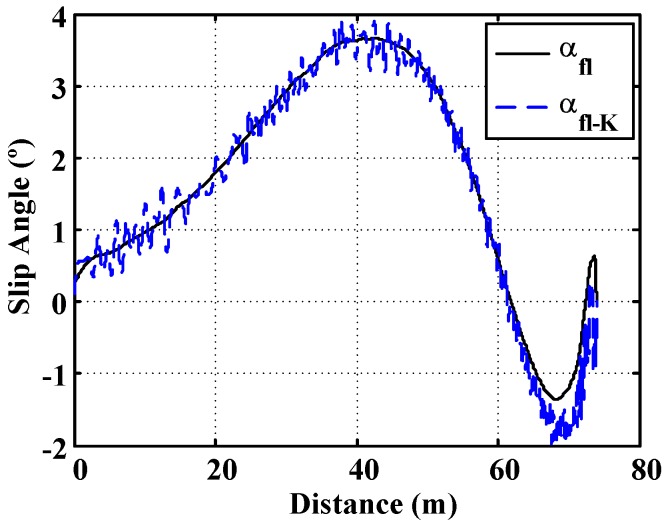
Curve simulation. Surface μ = 0.8. 500 m. radius curve. Slip angle comparison.

Figure 13 includes a comparison between the real values of the slip angle and the estimated values. This simulation shows that the algorithm is able to estimate the slip angle when a curved trajectory is carried out with the vehicle. 

**Figure 14:**
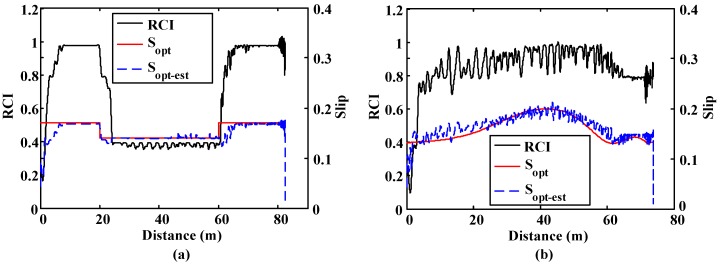
Road condition estimation and optimal slip comparison. (**a**) Straight line simulation. Surface transition: 20 m. on μ = 1, 40 m. on μ = 0.4 and 40 m. on μ = 1; (**b**) Curve simulation. Surface μ = 0.8. 500 m. radius curve.

Finally, the road condition and the optimal slip for both simulations are included in Figure 14. It can be seen that the road condition estimation algorithm can detect sudden changes in the adhesion condition. The algorithm yields a value representative of the surface in all road conditions, adapting its output quickly when an adhesion change occurs. Besides, the algorithm is also able to detect the road condition while cornering. In both simulations, the output of the ANN, *i.e.*, the optimal slip, is perfectly estimated. In both cases, the estimated optimal slip (S_opt-est_) matched the real optimal slip (S_opt_) perfectly.

### 6.3. Fuzzy Logic Estimation Algorithm Comparative

These simulations are included to compare the output of the proposed fuzzy block *vs.* a fuzzy road condition estimation block in which the slope of the µ–s curve is not included to detect the road condition. In the first simulation, the vehicle starts on a high-adhesion surface (μ = 1) and, after travelling 20 m on it, it moves to a low-adherence surface (μ = 0.8) for 20 m. Finally, the vehicle moves back to the high-adherence surface. 

**Figure 15:**
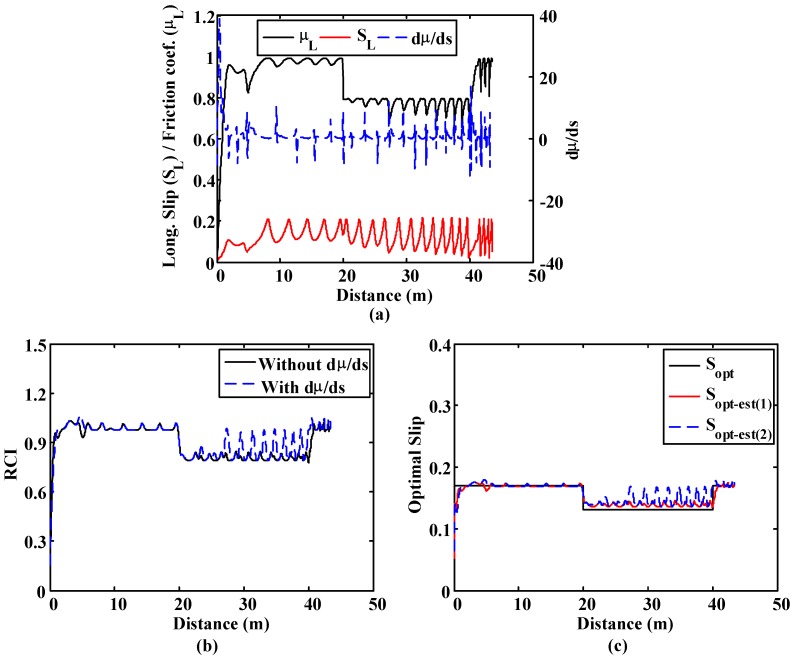
Straight line simulation. Surface transition: 20 m. on μ = 1, 20 m. on μ = 0.4 and 40 m. on μ = 1. (**a**) Friction coefficient, slip and dμ/ds; (**b**) Road condition fuzzy block outputs comparative; (**c**) Optimal slip comparative.

Figure 15a includes the inputs of both fuzzy logic blocks, which are the friction coefficient, the slip and dµ/ds. As it can be seen in Figure 15b, both fuzzy blocks can detect the changes in the adherence conditions. However, the output of the fuzzy block that has the slope of the µ–s trajectory as input is more stable. The output of the second block is stable when the slip is high. However, when the slips are low, which happens occasionally in the zone corresponding to the lower adherence surface, the output has big fluctuations. These fluctuations affect the optimal slip estimation, as seen in Figure 15c.

In the second simulation, the vehicle starts on a very low-adhesion surface (μ = 0.2) and, after covering 20 m on it, it moves to a low-adherence surface (μ = 0.4) for 20 m. Finally, the vehicle moves back to the very low-adherence surface. In this simulation the brake force is low, which causes very low levels of wheel slip. 

**Figure 16:**
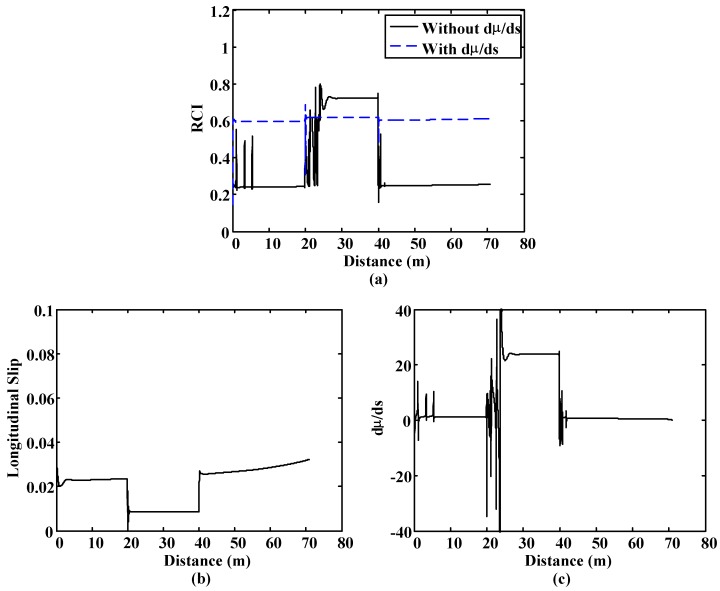
Straight line simulation. Surface transition: 20 m. on μ = 0.2, 20 m. on μ = 0.8 and 60 m. on μ = 0.2. (**a**) Road condition fuzzy block outputs comparative; (**b**) Slip; (**c**) dμ/ds.

Figure 16a shows the outputs of both fuzzy blocks. The fuzzy block that does not have dµ/ds as input is not capable of detecting the changes in the adherence of both surfaces due to the low slip values (Figure 16b). On the other hand, the output of the fuzzy block that has the slope of the µ–s trajectory as input (Figure 16c) detects the changes in the surface adequately, even with slip values below 0.02. 

**Figure 17:**
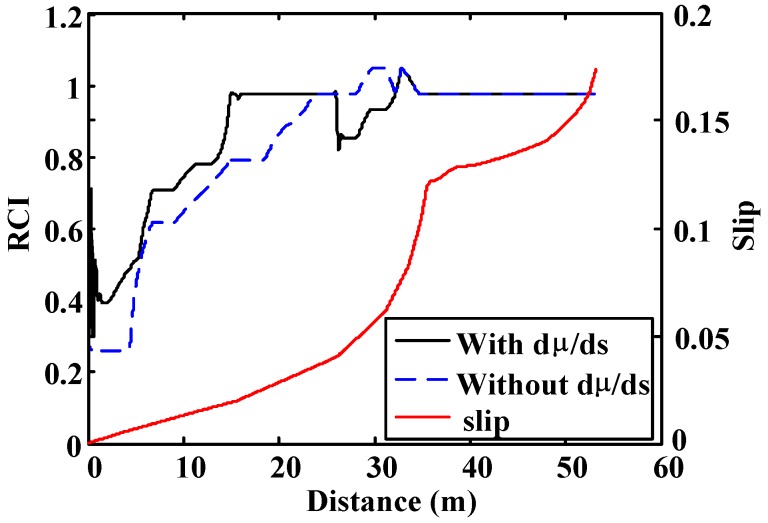
Straight line simulation. Surface μ = 1. Road condition fuzzy block outputs comparative.

Finally, in the third simulation the vehicle moves on a high-adhesion surface (μ = 1). The brake force is increased progressively, which causes a continuous increase in the wheel slip. As seen in Figure 17, the output of the fuzzy block that has the slope of the µ–s trajectory as input is faster in detecting the road condition. The second block needs slip values above 0.07 to provide correct values of the road condition. 

Simulation shows that the use of the rate of change of µ *vs.* s improves the estimation of the road condition when low slip conditions take place. Besides this, its use also increases the speed of the system to adapt its output at the beginning of the process and after sudden changes in road conditions. Finally, poor road condition detection affects optimal slip estimation since the output of the ANN depends on the reliability of the road condition estimation.

## 7. Experimental Results

**Figure 18:**
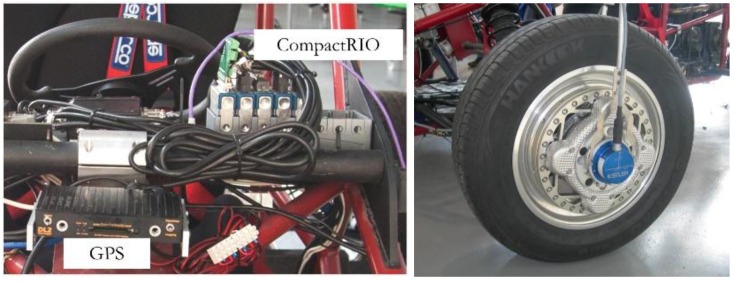
Data acquisition and control system (**Left**) and wheel force transducer (**Right**).

The performance of the proposed road condition detection system is evaluated using data acquired from a test vehicle. The vehicle was equipped with sensors to measure the variables involved in the process. The basic component of the data acquisition system was a programmable automation controller. The device selected was a CompactRIO 9074 system by National Instruments. Wheel forces were measured using a RoaDyn P625 wheel force transducer by Kistler. Figure 18 shows the main components of the data acquisition control systems and the wheel force transducer. 

**Figure 19:**
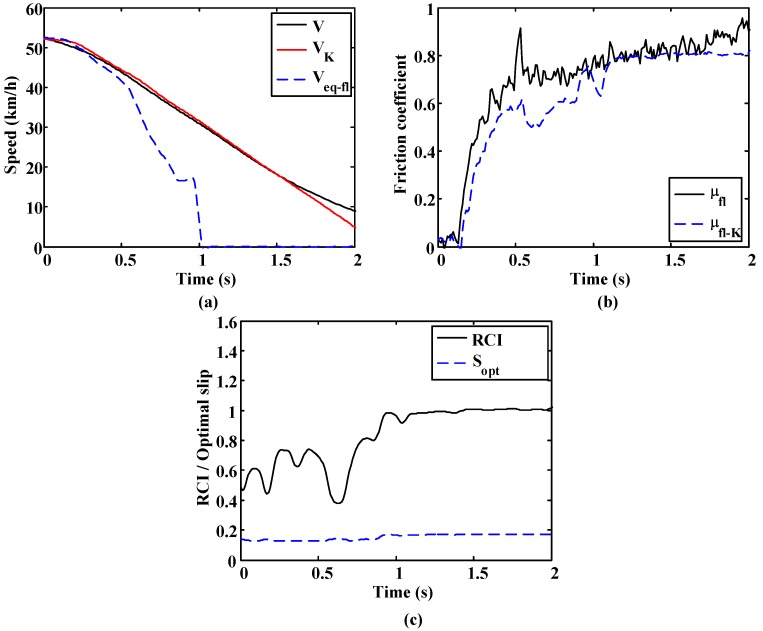
(**a**) Estimated and measured vehicle speed and wheel equivalent speed; (**b**) friction coefficient comparative; and (**c**) estimated road condition and optimal slip.

The following figures show the results from a test to verify the algorithm performance. This test focuses on the estimation of vehicle speed and the friction coefficient. Vehicle speed and friction coefficient are compared to the measurements obtained from the GPS sensor and the wheel force transducer, respectively. The estimated speed (V_k_) provided by the algorithm fits the speed measured by the GPS sensor correctly Figure 19a. The friction coefficient estimate (μ_K_) also shows an appropriate approximation to the real values Figure 19b. 

Straight-line brake tests were carried out on dry asphalt and wet asphalt. The brake pedal was progressively pressed until maximum brake pedal travel was reached, remaining in that position until the complete stop of the vehicle. 

**Figure 20:**
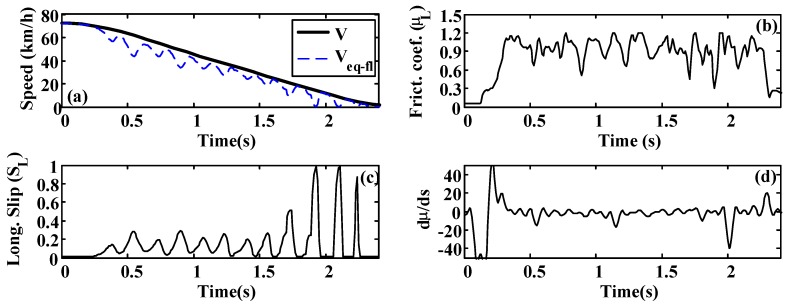
Dry asphalt. (**a**) Speed; (**b**) Friction coefficient; (**c**) Longitudinal slip; (**d**) Slope of µ–s curve.

**Figure 21:**
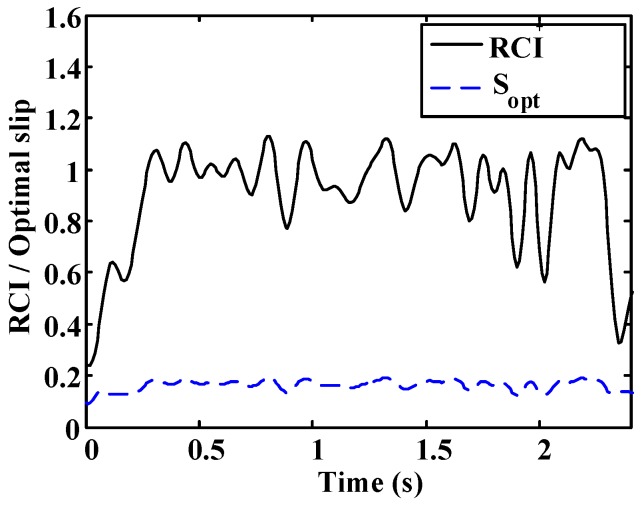
Dry asphalt: Estimated road condition and optimal slip.

**Figure 22:**
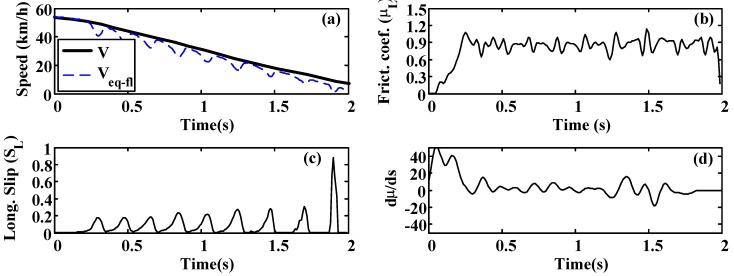
Wet asphalt. (**a**) Speed; (**b**) Friction coefficient; (**c**) Longitudinal slip; (**d**) Slope of µ–s curve.

**Figure 23:**
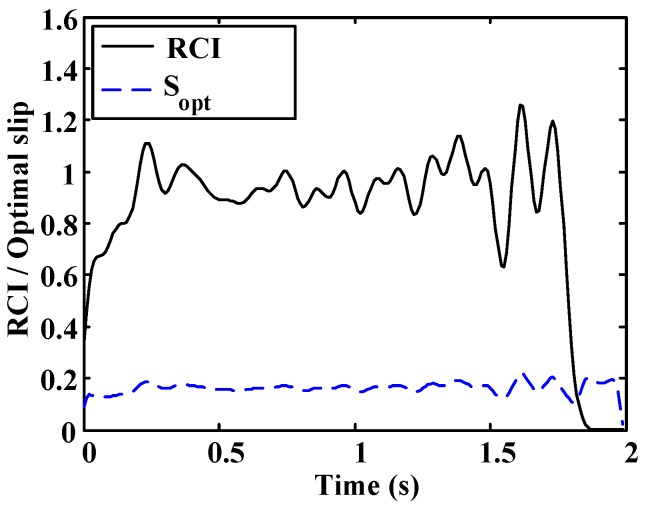
Wet asphalt: Estimated road condition and optimal slip.

A more oscillatory behavior is observed in the first test (Figure 20 and Figure 21). In both cases, the road condition estimation shows similar values, with slightly lower road condition estimates in the second test (Figure 22 and Figure 23). However, the optimal slip estimates remain with low fluctuations in both cases and with values around 0.17, which are representative of high adhesion surfaces.

**Figure 24:**
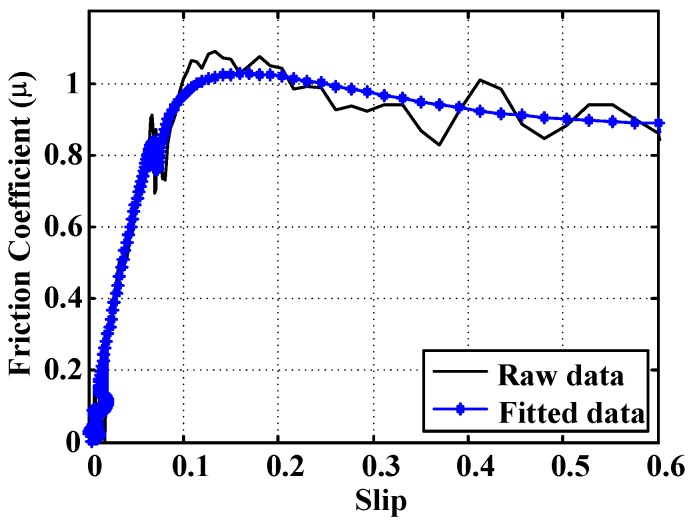
Experimental friction curve of the test surface.

A test to obtain the adhesion curve of the surface where the experiments were carried out was performed. The vehicle was equipped with the wheel force transducer. The surface was dry asphalt. Figure 24 shows the obtained raw data and the fitted data.

As it can be seen, the optimal slip for this surface is within the range 0.15–0.18, which coincides with the value provided by the ANN in the tests carried out on dry asphalt.

## 8. Conclusions

At present, different active safety systems are installed in most vehicles to make them more secure and reliable. Studies are continuously conducted to improve these systems. A fundamental premise of these studies is to have a reduced cost of implementation. All these active safety systems need specific information to work properly. This information is supplied by sensors, but there are variables that cannot be measured without incurring additional production costs on the vehicle. This work presents a parameter estimation algorithm to obtain the slip and the friction coefficient and, from these data, the road condition and the optimal slip of the surface where the vehicle is circulating. These parameters are fundamental for active safety systems or for automatic driving controls to be able to react faster and more smoothly to unexpected, dangerous situations. The proposed system obtains information from standard sensors installed vehicles. The aforementioned friction characteristics are obtained by means of parameter estimation techniques, extended Kalman Filter, fuzzy logic and neural networks.

Simulations have been carried out to evaluate the performance of the proposed system. These simulations show the ability of the proposal to yield accurate values of the road condition index and optimal sliding when changes have been made in the adhesion characteristics of the road condition in both straight and curved trajectories. The results provided by a fuzzy estimation block which has the slope of the friction *vs.* slip evolution as input and another block without that information have been compared. It has been verified that better results are obtained when the slope is taken into account, especially in areas of low slip, which corresponds to normal, non-aggressive driving. This is a main advantage of this proposal, since it shows that the proposed system is capable of obtaining the friction characteristics of the road when driving with low slip, being faster and more reliable in the estimation of the road condition compared to systems in which the slope of the friction curve is not used as input.

Experimental tests were carried out using a test vehicle. The results obtained show that our proposed system is able to detect the friction characteristics of the surface. Some encouraging results have been obtained to date. These values verify the good results obtained in simulations and the potential of this proposal. However, there is still a great deal of research to be carried out. Experimental tests have to be carried out to verify the aptitude of this proposal, especially in low and very low adherence conditions. The work reported here is exploratory. Future works will include tests on different surfaces and with different maneuvers.

## References

[1] Gray A., Ali M., Gao Y., Hedrick K., Borrelli F. Integrated Threat Assessment and Control Design for Roadway Departure Avoidance. Proceedings of the 15th International IEEE Conference on Intelligent Transportation Systems.

[2] Casselgren J., Sjödahl M., LeBlanc J. (2007). Angular spectral response–from covered asphalt. Appl. Optics.

[3] Bachmann T. (1995). The Importance of Thentegration of Road, Tyre and Vehicle Technology.

[4] Yamada M., Ueda K., Horiba I., Tsugawa S., Yamamoto S. (2005). Road surface condition detection technique based on image taken by camera attached to vehicle rearview mirror. Rev. Automot. Eng..

[5] Viikari V.V., Varpula T., Kantanen M. (2009). Road-Condition Recognition Using 24-GHz Automotive Radar. IEEE Trans. Intell. Transp. Syst..

[6] Breuer B., Bartz M., Karlheinz B., Gruber S., Semsch M., Strothjohann T., Xie C. The mechatronic vehicle corner of Darmstadt University of Technology- Iteration and cooperation of a sensor tire, new low-energy disc brake and smart wheel suspension. Proceedings of the FISITA 2000.

[7] Singh K.B., Arat M.A., Taheri S. (2013). An intelligent tire based tire-road friction estimation technique and adaptive wheel slip controller for antilock brake system. J. Dyn. Syst. Meas. Control.

[8] Edelmann J., Gobbi M., Mastinu G., Ploechl M., Previati G. (2015). Friction estimation at tire-ground contact. SAE Tech. Pap..

[9] Eichhorn U., Roth J. (1992). Prediction and Monitoring of Tyre/Road Friction.

[10] Umeno T., Ono E., Asano K., Ito S., Tanaka A., Yasui Y., Sawada M. (2002). Estimation of tire-road friction using tire vibration model. SAE Technical Paper.

[11] Schmeitz A., Alirezaei M. Model-based analysis of wheel speed vibrations for road friction classification using MF-Swift. Proceedings of the 4th International Tyre Colloquium, University of Surrey.

[12] Gustafsson F. (1997). Slip-based estimation of tire-road friction. Automatica.

[13] Carlson C.R., Gerdes J.C. (2005). Consistent nonlinear estimation of longitudinal tire stiffness and effective radius. IEEE Trans. Control Syst. Technol..

[14] Hahn J.O., Rajamani R., Alexander L. (2002). GPS-based real-time identification of tire-road friction coefficient. IEEE Trans. Control Syst. Technol..

[15] Lee C., Hedrick K., Yi K. (2004). Real-time slip-based estimation of maximum tire-road friction coefficient. IEEE/ASME Trans. Mechatron..

[16] Rajamani R., Phanomchoeng G., Piyabongkarn D., Lew J.Y. (2012). Algorithms for real-time estimation of individual wheel tire-road friction coefficients. IEEE/ASME Trans. Mechatron..

[17] Ray L.R. (1997). Nonlinear tire force estimation and road friction identification: Simulation and experiments. Automatica.

[18] Cabrera J.A., Ortiz A., Castillo J.J., Simón A. (2005). A Fuzzy logic control for antilock braking system integrated in IMMa tyre test bench. IEEE Trans. Vehicular Technol..

[19] Chen Y., Wang J. (2011). Adapative vehicle speed control with input injections for longitudinal motion independent road frictional condition estimation. IEEE Trans. Vehicular Technol..

[20] Choi M., Oh J.J., Choi S.B. (2013). Linearized recursive least squares methods for real-time identification of tire-road friction coefficient. IEEE Trans. Vehicular Technol..

[21] Erdogan G., Alexander L., Rajamani R. (2010). Adaptive vibration cancellation for tire-road friction coefficient estimation on winter maintenance vehicles. IEEE Trans. Control Syst. Technol..

[22] Castillo J.J., Cabrera J.A., Guerra A.J., Simon A. (2015). A novel electro-hydraulic brake system with tire-road friction estimation and continuous brake pressure control. IEEE Trans. Ind. Electron..

[23] Doumiati M., Victorino A., Charara A., Lechner D. (2009). Lateral load transfer and normal forces estimation for vehicle safety: Experimental test. Vehicle System Dynamics.

[24] Burckhardt M., Reimpell J. (1993). Fahrwerktechnick: Radschlupf-Regelsysteme.

[25] Wenzel T.A., Burnham K.J., Blundell M.V., Williams R.A. (2006). Dual extended Kalman filter for vehicle state and parameter estimation. Vehicle Syst. Dyn..

[26] Kim J. (2009). Identification of lateral tyre force dynamics using an extended Kalman filter from experimental road test data. Control Eng. Pract..

[27] Khanesar M.A., Kayacan E., Teshnehlab M., Kaynak O. (2012). Extended Kalman Filter Based Learning Algorithm for Type-2 Fuzzy Logic Systems and Its Experimental Evaluation. IEEE Trans. Ind. Electron..

[28] Guo H., Chen H., Xu F., Wang F., Lu G. (2013). Implementation of EKF for Vehicle Velocities Estimation on FPGA. IEEE Trans. Ind. Electron..

[29] Ray L.R. (1995). Nonlinear State and Tire Force Estimation for Advanced Vehicle Control. IEEE Trans. Control Syst. Technol..

